# Influence of Nutritional Strategies on Performance, Gut Barrier Function and Microbiota Composition in Weaned Piglets

**DOI:** 10.3390/ani15233422

**Published:** 2025-11-27

**Authors:** Sara Isusi, Guillermo Usero-Alonso, Jose Alberto Murillo, Ana Belén Gonzalez-Guijarro, Antonio Muñoz, Guillermo Ramis

**Affiliations:** 1 R&D Department, FarmFaes—Ingaso Farm, Laciego, 01308 Vitoria, Spain; gusero@farmfaes.com (G.U.-A.); jmurillo@farmfaes.com (J.A.M.); 2 Departamento de Produccion Animal, Facultad de Veterinaria, Universidad de Murcia, 30100 Murcia, Spain; abgg@um.es (A.B.G.-G.); antmunoz@um.es (A.M.); guiramis@um.es (G.R.); 3 Instituto Murciano de Investigación Biomedica Pascual Parrilla, 30120 Murcia, Spain

**Keywords:** piglet, weaning, gut microbiota, intestinal integrity, growth performance

## Abstract

The restriction of antibiotics and therapeutic zinc oxide in piglet diets has increased challenges related to gut health and post-weaning performance. This study assessed the effects of a diet supplemented with organic acids and the probiotic *Clostridium butyricum* on intestinal integrity, fecal microbiota composition, and performance parameters in weaned piglets. Forty piglets were selected at weaning, and fecal samples were collected on day 0 and day 14 of supplementation. Gene expression of tight junction proteins (zonulin, occludin) and inflammatory markers (calprotectin, IFN-γ, TGF-β) was analyzed by PCR. Bacterial DNA quantification was used to evaluate microbiota changes. In addition, average daily gain (ADG), feed conversion ratio (FCR), and mortality were recorded across three feeding phases: CONTROL, TRANSITION, and 0M. No significant changes were found in tight junction protein expression post-weaning, but reductions in IFN-γ and TGF-β suggest improved immune modulation. Piglets in the TRANSITION and 0M groups showed higher ADG (CONTROL = 292.28 g/day; TRANSITION = 300.09 g/day; 0M = 307.45 g/day) and reduced mortality (CONTROL = 6.48%; TRANSITION = 5.10%; 0M = 5.08%) compared to CONTROL. These findings indicate that targeted dietary supplementation can support gut health and performance in weaned piglets, offering a promising alternative to medicated feed under current regulatory constraints. Further research is guaranteed to refine these strategies for broader application in sustainable pig production.

## Simple Summary

Weaning is a stressful moment for piglets that often causes digestive problems, poor growth, and increased mortality. Traditionally, antibiotics were used in feed to control these issues, but recent regulations restrict their use. As a result, the pig industry is searching for natural alternatives to protect the gut health of young pigs. In this study, a gut health-promoting additive was tested in the feed of piglets at post-weaning to see if it could support the animals during this critical period. Changes in gut health and intestinal inflammation were measured through fecal samples, and pig growth and survival were monitored over time. Results showed that while the gut barrier did not change significantly, signs of inflammation decreased. Piglets in the supplemented diet also grew better and had lower death rates than those on a standard diet. These findings suggest that targeted nutritional strategies may help piglets stay healthy without relying on banned substances. This could be beneficial to more sustainable pig production under current legal restrictions.

## 1. Introduction

Commercial weaning usually takes place around 21–28 days of age in piglets, and it is recognized as a critical period that induces stress and leads to abrupt changes in the gut, which can negatively affect growth performance and increase the risk of health issues, such as diarrhea [1].

The intestinal epithelium plays a critical role in maintaining the barrier function, which is essential to protect the organism from harmful substances while allowing nutrient absorption. This barrier is primarily established through tight junctions (TJs) and a dynamic interplay of cellular mechanisms that regulate permeability and repair. Tight junctions form a selective barrier that regulates paracellular permeability between the intestinal lumen and serosa and the pore size of the epithelium, known as ‘gate function’ [2]. Early weaning results in increased intestinal permeability and decreased expression of tight junction proteins in piglets [3]. Inflammatory markers also contribute to gut health assessment. Calprotectin is a crucial biomarker in the gut, primarily indicating inflammation and aiding in the diagnosis and management of various gastrointestinal diseases [4]. Meanwhile, cytokines, such as transforming growth factor β (TGF-β) and interleukins (IL) IL-1α and IL-6, are continuously expressed by the intestinal epithelium and may play a role in the basal flow of immune cells in the gut mucosa [5]. As well, elevated levels of pro-inflammatory cytokines correlate with structural changes in the intestinal mucosa, contributing to conditions like post-weaning diarrhea [6].

Another key aspect in maintaining piglet health is the intestinal microbiota, which plays an essential role in nutrient absorption, immune system development, and protection against pathogens, and it is determined by various factors, such as diet, age, stress and environment [1]. Bacterial colonization in the gut has profound effects on both the morphology of the intestine and the expression of inflammatory cytokines, which could have implications for understanding gut health and disease in pigs [7]. The development of the intestinal microbiota in piglets begins at birth when neonates are exposed to microorganisms through the vaginal canal and their immediate environment [8] and continues during lactation, closely resembling that of their mothers, preparing them to transition from milk to solid food. The presence of specific bacterial groups, like Bacteroidetes and Firmicutes, plays a crucial role in these metabolic processes, influencing the piglets’ growth and health [8]. There are some genera of bacteria identified as the “core” microbiome, consisting of 19 bacterial genera, including *Bacteroides*, *Prevotella* and *Lactobacillus*, which are present in over 90% of pigs across different ages, and they are crucial for the growth and gut health of the host [1].

With recent findings about post-weaning and the ban on high levels of antibiotics and zinc oxide (ZnO) in feed, there is a growing interest in nutritional alternatives [1], such as organic acids and probiotics. Acids are compounds typically classified as organic and inorganic and can improve growth by reducing or maintaining gastric pH, thereby increasing nutrient digestibility and limiting the growth of pathogenic bacteria. Combinations of organic acids are used commercially, as the response of the mixture of acids is better than using only one, due to the different properties of the acids throughout the gastrointestinal tract [9]. However, the effects of organic acids on gut microbiota composition are less clear, with some studies showing little impact on microbial structure. The controversy mainly stems from variable results, differences in product formulations, and the complexity of piglet gut health, making it challenging to predict outcomes in every setting [10].

On the other hand, probiotics have shown benefits such as reducing digestive disorders, improving growth, and supporting gut health, but research results are often inconsistent and vary between farms, likely due to differences in probiotic strains, dosages, and management practices [11]. *Clostridium butyricum* as a probiotic has been used in recent years, as it has been reported to influence the better growth of animals, improve immune response and regulate the structure and composition of the gut microbiota, as its addition to the diet leads to an increase in the height of the intestinal villi and a greater depth of the crypts [12].

This study aimed to evaluate a nutritional strategy for the gradual reduction in pharmacological levels of zinc oxide (ZnO) in nursery pig diets. The approach consisted of the three feeding phases with decreasing ZnO inclusion and the incorporation of gut health-promoting additives. This strategy was designed to support intestinal integrity, promote a stable microbiota transition, and ultimately enable the withdrawal of ZnO without compromising animal performance. We hypothesized that supplementing the diet with specific gut health additives would enhance intestinal integrity and microbiota stability during the weaning transition, thereby maintaining performance and reducing weaning-associated stress.

## 2. Materials and Methods

The methods of animal handling during research were developed by the ethical principles set out in the European Directive 2008/120/EC, published in the Official Journal of the European Union (2008) [13], and in the national legislation, as outlined in Royal Decree 1135/2002, published in the BOE (2002) [14], which establishes the minimum welfare requirements for pigs kept for commercial production, including housing conditions, feeding care, and management practices. These provisions were applied together with the updates and requirements introduced by Royal Decree 159/2023 [15]. Only non-invasive fecal sampling and routine performance monitoring were conducted, in accordance with current animal welfare legislation. The study also complied with the general principles of Royal Decree 53/2013 [16], although no experimental or invasive procedures requiring ethical approval were performed.

### 2.1. Experimental Design and Housing

The study was conducted under field conditions in four commercial nursery farms located in northeastern Spain. Data was collected across three defined time periods, each corresponding to a specific dietary treatment applied during the first two weeks post-weaning (days 1–14). From day 15 onwards, all piglets received the same basal diet until the end of the nursery stage. The three experimental periods were (1) CONTROL (3 January 2019–31 May 2021), where piglets received a standard initial feed (IF) supplemented with 3.1 kg/ton of ZnO; (2) TRANSITION (1 June 2021–26 June 2022), where the same IF was used but supplemented with a reduced dose of ZnO (2.1 kg/ton) and a gut health additive blend consisting of sorbic acid with an inclusion of 65 g/kg, benzoic acid with an inclusion of 165 g/kg and a probiotic (*Clostridium butyricum*) with an inclusion of 1.25 × 10^11^ UFC/kg; and (3) 0M (27 June 2022–24 June 2024), where the IF contained only the additive blend described above, with no ZnO supplementation.

During the 0M period, a subset of 10 clinically healthy weaned piglets (28 days old) was randomly selected from each farm (*n* = 40). Selection criteria ensured similar initial body weights and health status, representative of the entire population. All piglets were housed in conventional nursery facilities with 20 animals per pen and provided with ad libitum access to feed and water throughout the experimental period. Fecal samples were collected individually from the selected piglets on the day of weaning (day 0) and again on day 14 post-weaning for microbiota and intestinal health marker analyses.

### 2.2. Performance Data

Performance data were collected from a total of 491,000 piglets raised in four commercial nursery farms located in northeastern Spain between 2019 and 2024. Animals were distributed across different batches (*n* = 140), which served as the experimental unit for statistical analysis. The number of batches and average animals per batch per farm were as follows: Farm A: 30 batches, 5700 piglets per batch; Farm B: 35 batches, 3800 piglets per batch; Farm C: 40 batches, 3100 piglets/batch; and Farm D: 35 batches, 1800 piglets/batch.

Batches were assigned to one of the three experimental periods based on time of production and the diet applied during the initial nursery phase (days 1–14 post-weaning): (1) CONTROL, *n* = 174,000 piglets; (2) TRANSITION, *n* = 105,000 piglets; and (3) 0M, *n* = 212,000 piglets. For each batch, the following performance parameters were recorded: initial body weight (BW0), final body weight (BW1), average daily gain (ADG), feed conversion ratio (FCR) and mortality rate. The nursery cycle began at 28 ± 5 days of age with an average body weight of 5.5 ± 0.5 kg and ended at 44 ± 4 days of age, with a final body weight of 18 ± 2.7 kg.

### 2.3. Diets

After weaning, piglets received different initial diets during the first 14 days of the nursery phase, depending on the experimental period (CONTROL, TRANSITION or 0M). These diets were formulated with different levels of ZnO and inclusion or absence of gut health additives as part of the nutritional strategies under evaluation. None of the diets contained antibiotics. The formulation of each initial feed—including ingredient composition and estimated nutrient content—is detailed in Table 1.

Diets were analyzed for crude protein (CP), metabolizable energy (ME), standardized ileal digestible lysine (Lys SID), and Lys SID to net energy ratio (Lys SID/NE). Differences between diets were not statistically compared, as formulations were designed to be nutritionally equivalent except for the tested additives.

### 2.4. Animal Sampling

Fecal samples were collected aseptically and directly from the rectal ampoule of each piglet to prevent environmental contamination. Each fecal sample was split into two 100 mg portions and placed into 1.5 mL sterile Eppendorf tubes (Eppendorf Canada Ltd., Mississauga, ON, Canada), snap-frozen in liquid nitrogen, and stored at −80 °C until further analysis. One sample was used for microbiota analysis, while the second was used for gut health biomarker analysis, including markers of intestinal integrity and inflammation.

### 2.5. Microbiota Composition Analysis—16S rRNA Isolation

Fecal samples were analyzed at the Instituto Murciano de Investigación Biosanitaria Pascual Parrilla, El Palmar (Murcia), España, using high-throughput sequencing of the 16S rRNA gene to assess microbial composition and diversity. This technique enables identification and characterization of bacterial taxa without the need for culturing. Taxonomic assignments are possible due to the presence of hypervariable regions (V1–V9) containing sufficient sequence diversity to classify microorganisms. A single-step qPCR methodology combining amplification of the region of interest together with the addition of barcodes and Illumina adapters has been used. Real-time PCR allows absolute quantification of the copy number by means of a standard curve. DNA extraction and library preparation were performed using the “Quick-16S Plus NGS Library Prep Kit (V3–V4, UDI)” (Zymo Research, Irvine, CA, USA), which targets the V3–V4 hypervariable regions of the 16S rRNA gene. The primers used for amplification were:

- Forward: 341f (CCTACGGGDGGCWGCAG, CCTAYGGGGYGCWGCAG).
- Reverse: 806r (GACTACNVGGGTMTCTAATCC).

For each reaction, 1–10 ng of extracted DNA was used. A single-step qPCR protocol was applied, combining target amplification, barcode addition, and adapter ligation. This produced amplicons of approximately 606 bp, which were pooled in equal volumes and purified using magnetic bead-based cleanup, following the manufacturer’s instructions. Final libraries were sequenced on the NextSeq 2000 platform (Illumina, San Diego, CA, USA) using paired-end sequencing (2 × 300 bp). The analysis yielded the relative abundance of bacterial taxa at the family, genus, and species levels. Taxonomic classification was based on sequence comparison within the conserved and variable regions of the 16S rRNA gene.

### 2.6. Sequencing of Intestinal Integrity Markers

Gene expression analysis was conducted to evaluate the markers related to intestinal barrier function and immune response. The target genes included two tight junction (TJ) proteins: occludin (OCL) and zonulin1 (ZON); an indicator of the presence of inflammatory cells: calprotectin S100 (CAL); and two cytokines representative of inflammatory (IFNγ) and anti-inflammatory (TGF-β) activity. Reverse transcription and quantitative PCR (qPCR) were performed to quantify mRNA expression levels of each gene. Primers specific to each gene are detailed in Table 2. All qPCR reactions were conducted in triplicate to ensure data reliability and reproducibility.

### 2.7. Bioinformatics and Statistical Analysis

Performance data were analyzed using XLSTAT (v2023.1.6.1410, Addinsoft, Denver, CO, USA) in Microsoft Excel. Descriptive statistics (means ± standard error of the mean, SEM) were calculated for each performance parameter. Comparisons between experimental periods were made using one-way ANOVA. Significance was set at *p* < 0.05.

For microbiota analysis, raw sequencing data were processed following the standard workflow of the institutional Bioinformatics Platform. Primary FASTQ files were generated using the Dragen BCL Convert V12.7.4 pipeline, Illumina, San Diego, CA, USA, and subsequently transferred to the platform for downstream analysis, which included quality filtering, denoising and chimera removal. Taxonomic assignment of amplicon sequence variants (ASVs) was performed using the SILVA reference database (March 2020). In addition, relative numbering was carried out using a real-time PCR standard curve generated from serial dilutions of the ZymoBIOMICS^TM^, Irvine, CA, USA 16S qPCR Standard (7.5 × 10^6^ copies/µL). Bacterial families, genera, and species were ranked by relative abundance, and taxa representing ≥1% of total relative abundance in at least one sample were considered predominant for downstream analyses. Differences in the relative abundance of bacterial taxa between pre-weaning and post-weaning samples were analyzed using a one-way ANOVA in XLSTAT (v2023.1.6.1410, Addinsoft, Denver, CO, USA), with sampling time (day 0 vs. day 14 post-weaning) as the fixed factor. Statistical significance was set at *p* < 0.05.

For intestinal integrity markers, differences between time points (weaning vs. day 14 post-weaning) were analyzed by paired *t*-test or repeated measures ANOVA, as appropriate. Principal Component Analysis (PCA) was used to visualize clustering patterns based on gene expression data.

Finally, Pearson’s correlation analysis was used to assess the relationship between bacterial relative abundance at the genus level and the gene expression levels of intestinal integrity and immune markers. Correlation coefficients (ρ) were calculated, and associations were considered statistically significant at *p* < 0.05.

## 3. Results

### 3.1. Productive Data

Table 3 below shows the results of the performance data between the three periods. It can be observed that the initial body weight (BW0) remained stable over the three periods. Final body weight (BW1) and average daily gain (ADG) were significantly lower (*p* < 0.05) in CONTROL than in TRANSITION or the 0M period. Feed conversion ratio (FCR) and % mortality were significantly (*p* < 0.05) higher in CONTROL than in TRANSITION or the 0M period.

### 3.2. Evolution of the Fecal Microbiota After Weaning

Table 4 shows the differences in relative abundance of the bacterial species studied between pre-weaning and post-weaning. *Lactobacillus reuteri*, [*Eubacterium*] *biforme*, *Ruminococcus bromii*, and *Desulfovibrio piger* increased significantly (*p* < 0.05). *Bilophila wadsworthia*, *Eubacterium coprostanoligenes*, *Ruminococcus faecis*, and *Alistipes shahii* decreased significantly after weaning (*p* < 0.05).

**Figure 1:**
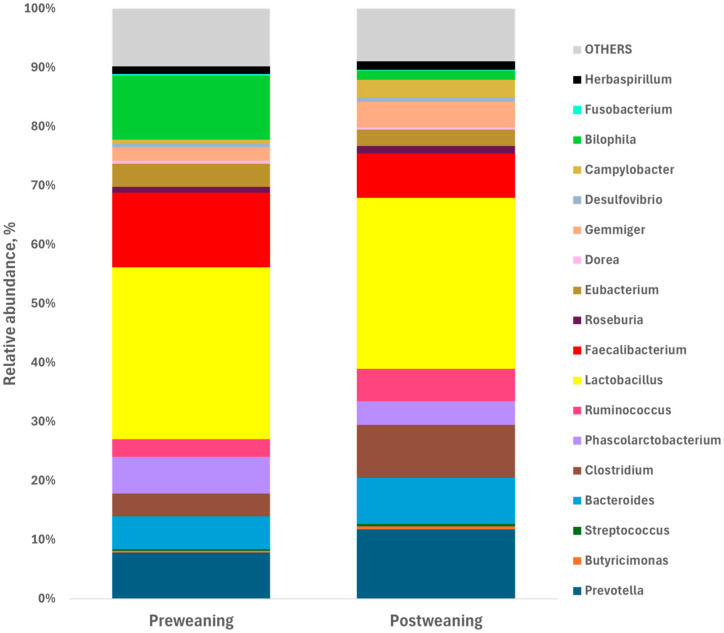
Relative abundance of the main bacterial genera identified in fecal samples collected from piglets at day 0 (pre-weaning) and day 14 (post-weaning) during the 0M period. Bars represent the mean relative abundance (%) of the most predominant genera (≥1% of total sequences in at least one group).

The results of the relative abundance of bacterial genera between pre-weaning and post-weaning are shown in Figure 1, where *Prevotella*, *Ruminococcus*, *Clostridium*, and *Lactobacillus* increased during the 14 days post-weaning, while others, such as *Bilophila* and *Faecalibacterium*, decreased.

### 3.3. Evolution of the Intestinal Integrity After Weaning

**Figure 2:**
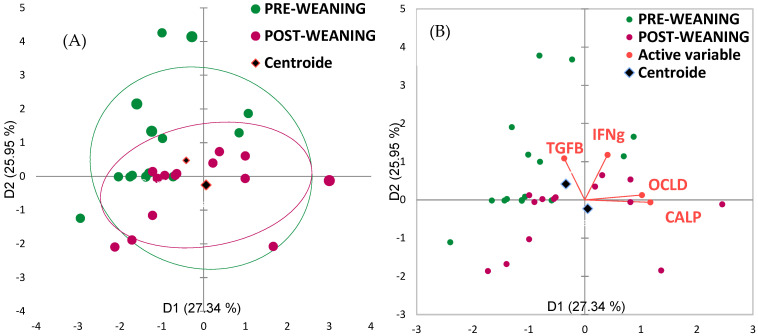
Principal component analysis (PCA) of intestinal integrity and inflammatory markers in piglets at day 0 (pre-weaning) and day 14 (post-weaning) during the 0M period. (**A**) PCA score plot showing the distribution of individual samples based on gene expression profiles of tight junction (TJ) proteins and inflammatory cytokines. (**B**) PCA biplot indicating the contribution of each marker to the separation between groups.

PCA of all intestinal integrity showed a clear separation between them, with IFN-γ and TGF-β clustering in the pre-weaning zone on the one hand and calprotectin and occludin towards the post-weaning zone on the other, with two principal components explaining 27.34% of the variation in Figure 2.

Table 5 shows the mean expression results of the gut integrity markers and the differences between pre-weaning and post-weaning. The two factors that have a significant difference are INF-γ and TGF-β (*p* < 0.05).

### 3.4. Correlation Between Intestinal Integrity and Fecal Microbiota

It can be seen from the results of the correlations between bacterial species and the markers of intestinal integrity studied in Table 6 that *Eubacterium biforme* has a significant (*p* < 0.010) negative correlation with calprotectin, while *Roseburia faecis* has a positive correlation; *Lactobacillus reuteri* has a negative correlation with occludin, while *Desulfovibrio piger* has a significant (*p* < 0.05) positive correlation; *Faecalibacterium prausnitzii* has a significant (*p* < 0.05) positive correlation with zonulin; *Bilophila wadsworthia* has a positive correlation with IFN-γ; and *Ruminococcus faecis* has a significant (*p* < 0.01) positive correlation with TGF-β.

## 4. Discussion

For decades, post-weaning enteric disease prevention relied on the use of ZnO and antibiotics. However, their restriction in the EU, including the ban of ZnO at therapeutic levels since June 2022 [20,21], has declined nursery performance, worsened post-weaning enteric diseases, increased mortality, and even aggravated common pathologies such as streptococcosis due to deterioration in gut integrity [22,23]. This study presents a transition strategy until the total elimination of ZnO, incorporating a probiotic and a blend of organic acids. Results showed improved performance rather than decline. Different studies confirm probiotics’ benefits, with *B. subtilis* achieving similar ADG and FCR compared to those obtained with therapeutic doses of ZnO [24]. Furthermore, using a probiotic mix of *C. butyricum*, *B. subtilis* and *B. licheniformis* enhanced ADG and FCR, matching antibiotic-treated groups [10]. Using *C. butyricum* alone also improved ADG, with even better results when combined with *B. licheniformis* [25]. Conversely, another study found no significant ADG differences but reported a 200 g/kg FCR increase [26]. Our study demonstrated that the combination of ZnO reduction and probiotic supplementation improves the performance data. Moreover, this improvement persists over time even after ZnO withdrawal, suggesting a shift in the farm microbiota.

In the study, we found that weaning leads to some changes in microbiota composition. At weaning, the families with the highest relative abundance in the samples were *Enterobacteriaceae*, *Erysipelotrichaceae*, *Lactobacillaceae* and *Ruminococcaceae*. These findings differ from previous studies that reported alternative profiles for the same period [27]. These differences suggest that the composition of the microbiota before weaning is influenced by various factors such as sow nutrition and health status, piglet management, and environmental conditions [28]. At the genus level, the most abundant taxa in our study at weaning were *Lactobacillus*, *Faecalibacterium*, *Bilophila*, *Prevotella*, *Phascolarctobacterium*, and *Bacteroides*. This pattern aligns with studies reporting *Lactobacillus*, *Bacteroides* and *Streptococcus* as dominant genera during lactation [29], due to their ability to utilize a wide range of milk oligosaccharides [30].

Notably, certain bacterial genera, such as *Lactobacillus* and *Bacteroides*, persisted after weaning. In particular, the genus *Lactobacillus* plays a crucial role in promoting intestinal health by regulating immune-related pathways, especially the IgA-mediated intestinal immunity network [31]. Its persistence may be attributed to the presence of organic acids in the diet, which promote growth by reducing or establishing gastric pH, increasing proteolysis and nutrient absorption, and limiting the growth of pathogenic bacteria [32]. Beyond acidification, additional mechanisms may explain the resilience of *Lactobacillus* after weaning. First, several *Lactobacillus* species can degrade complex polysaccharides, allowing adaptation to the cereal-based solid diets rich in starch and non-digestible carbohydrates that piglets receive post-weaning [33]. Second, during the early post-weaning period, the delayed maturation of host pancreatic α-amylase results in undigested starch reaching the lower intestine, providing an accessible substrate for *Lactobacillus* and other saccharolytic bacteria [34]. And third, *Lactobacillus* is consistently reported as a part of the core porcine microbiome across suckling, nursery, and grow-finishing stages, being consistently involved in carbohydrate fermentation [35]. These processes, together with the observed increase in species of *Lactobacillus* such as *L. reuteri*, suggest the establishment of a healthier, low-pH microbial environment after dietary transition.

Changes in microbiota composition not only impact microbial colonization but also play a crucial role in shaping intestinal morphology and modulating cytokine expression [7]. In the present study, no significant differences were observed in the expression of tight junction (TJ) proteins, and the calprotectin concentrations remained stable between days 0 and 14. Although these results contrast with reports of early post-weaning TJ disruption [2], they may reflect improved microbial resilience in our system. The observed increase in *L. reuteri*—associated with lactate production, reduced luminal pH and enhanced colonization resistance—supports this interpretation and may indicate a more balanced microbial environment during the transition.

Beyond these genus-specific associations, microbial metabolites act as a key mechanistic link between microbiota composition and intestinal integrity. SCFA-producing bacteria such as *Faecalibacterium*, members of *Ruminococcaceae*, *Oscillibacter* or certain *Lachnospiraceae* generate acetate, propionate and butyrate, which are essential for epithelial physiology. Butyrate serves as the primary energy source for colonocytes, promotes epithelial repair, and strengthens barrier function by enhancing TJ protein assembly [36]. It also exerts anti-inflammatory effects by increasing regulatory T cell activity and suppressing pro-inflammatory cytokine signaling. Likewise, lactate-producing bacteria such as *Lactobacillus* help maintain a low gut pH and inhibit the growth of opportunistic pathogens, supporting epithelial stability during the stressful post-weaning phase [37]. Furthermore, commensal bacteria play a central role in colonization resistance. After weaning, dysbiosis typically leads to an expansion of *Enterobacteriaceae* and other facultative anaerobes, which is associated with intestinal inflammation and post-weaning diarrhea [22]. In contrast, beneficial taxa like *Bacteroides*, *Ligilactobacillus salivarus*, and *Lactobacillus amylovorus* contribute to immune stimulation [38], competitive exclusion of pathogens, and maintenance of barrier integrity. The presence of anti-inflammatory genera such as *Faecalibacterium* before weaning has been proposed as a protective factor against post-weaning dysbiosis [33], suggesting that a more mature and SCFA-rich microbiota may help mitigate inflammatory responses after transition to solid feed.

High fecal calprotectin is linked to dysbiosis, marked by increased pro-inflammatory microbiota and reduced short-chain fatty acid-producing bacteria [39]. In this study, species from the genus *Bacteroides* that produce medium-chain fatty acids (MCFAs) exhibited a positive correlation with elevated calprotectin levels. This finding may be explained by the role of certain species, such as *Bacteroides fragilis*, which plays a role in maintaining eubiosis in the gastrointestinal tract through MCFA production. However, *B. fragilis* can also act as an opportunistic pathogen under conditions of intestinal dysbiosis, highlighting its dual role in gut health [40]. Altogether, these results support the idea that the stability of physiological markers observed in our study may be partly explained by the persistence or functional compensation of beneficial microbial populations such as *L. reuteri* or *Eubacterium* capable of producing SCFAs, lactate, and other regulatory metabolites. These microbial features appear to support epithelial barrier maintenance and limit inflammatory activation during the post-weaning period.

Significant differences were observed in the levels of IFN-γ and TGF-β, with both showing decreased expression in fecal samples after weaning. Previous studies have reported that IFN-γ expression increases up to 15 days post-weaning, indicating a prolonged immune response, while TGF-β expression in the gut tends to decline during this period [41]. These changes, nonetheless, appear to be transient [42]. In this study, we also observed positive correlations between the expression of TGF-β and bacterial species such as *Ruminococcus faecis*. This finding suggested that TGF-β may play a pivotal role in maintaining intestinal structure and function during the weaning process [42], as this bacterial species belongs to a genus known for its contribution to short-chain fatty acid (SCFA) production. PCA supported these findings, showing that IFN-γ and TGF-β expression were linked to the pre-weaning phase, where immune activation and stress enhance the adaptive response and gut microbiota maturation, key for immune function [43]. In contrast, occludin expression is associated with the post-weaning samples, suggesting a potential role in the repair of the intestinal epithelium following weaning [44]. Other authors, however, have found that occludin expression decreases 14 days after weaning, indicating that the intestinal barrier is not yet repaired and intestinal permeability is increased [2]. The initial findings regarding the gut health additive are promising; however, further research is essential to explore its implications and potential applications across diverse contexts. A deeper understanding of how variables such as diet, environment and sanitary conditions interact with gut health additives will be crucial for optimizing their use.

The authors are aware that the main limitation of this study is the lack of microbiota data for the CONTROL and TRANSITION periods. However, the decision to conduct this study was based on the observation that the performance data improved during the TRANSITION period and persisted even after ZnO removal.

## 5. Conclusions

In the present study, the results show that gradual reduction of zinc oxide, combined with organic acids and *Clostridium butyricum*, can support a stable and functionally resilient intestinal ecosystem during the post-weaning transition. This strategy did not compromise performance under commercial conditions, likely because the acid blend promoted a lower intestinal pH that favored beneficial bacteria such as *Lactobacillus* and *Bacteroides*, while the increase in *Eubacterium* and *Ruminococcus* suggests enhanced production of butyrate and other short-chain fatty acids essential for epithelial repair and anti-inflammatory balance. The inclusion of *C. butyricum* further strengthened microbial stability by competing with opportunistic pathogens and contributing to colonization resistance. Together, these microbial adaptations may explain the absence of changes in tight junction expression and calprotectin levels, indicating preserved barrier function. Thus, the nutritional approach tested here appears to offer a biologically robust alternative to pharmacological ZnO, supporting intestinal health, maintaining growth, and contributing to more sustainable pig production.

## Figures and Tables

**Table 1:** Initial feed diets supplied to piglets for each experimental period from weaning to day 14. Values indicate the ingredient composition and nutritional content of the diets provided during each trial.

Ingredients, %	CONTROL	TRANSITION	0M
Wheat	29.99	29.99	29.99
Maize	17.00	24.80	25.00
Barley	15.00	15.00	15.00
Soybean 48	14.00	5.30	5.30
Full-fat soybean	4.00	4.00	4.00
Mineral and vitamin premix	4.00	4.00	4.00
Soybean oil	2.18	1.92	1.92
Wheat bran	2.00	2.00	2.00
Blood meal	2.00	2.00	2.00
Whey powder fat-filled	1.80	1.80	1.80
Fish oil	1.25	1.25	1.25
Whey powder	1.25	1.25	1.25
Beetroot pulp	1.00	1.00	1.00
Sacarose	1.00	1.00	1.00
Monocalcium phosphate	0.85	0.90	0.90
Calcium carbonate	0.72	0.76	0.76
Salt	0.50	0.50	0.50
Sodium bicarbonate	0.32	0.33	0.33
Zinc oxide	0.31	0.20	-
Lysine	0.27	0.54	0.54
Threonine	0.23	0.35	0.35
Methionine	0.20	0.29	0.29
Tryptophan	0.09	0.13	0.13
Valine	0.05	0.20	0.20
Blend of organic acids		0.30	0.30
*Clostridium butyricum* UFC/g		12.5 × 10^10^	12.5 × 10^10^
Chemical composition	CONTROL	TRANSITION	0M
Crude protein, %	19.1	16.0	16.0
M. E., Kcal/kg	3342.0	3332.0	3332.0
Lysine SID	1.3	1.3	1.3
LysSID/NE, g/MKcal	5.2	5.1	5.1

**Table 2:** Primers for tight junction (TJ) proteins, cytokines, and inflammatory indicators used in this study. The table lists the gene name and primer sequences (forward and reverse) employed for quantitative real-time PCR (qPCR) analysis.

Gene	Forwerd Primer	Reverse Primer	Reference
Calprotectin (S100 calcium binding protein A8)	5′-AATTACCACGCCATCTACGC-3′	5′-TGATGTCCAG CTCTTTGAACC-3′	[17]
Occludin	5′-TTGCTGTGAAA ACTCGAAGC-3′	5′-CCACTCTCTCCGCATAGTCC-3′	[17]
Zonulin 1	5′-CACAGATGCC ACAGATGACAG-3′	5′-AGTGATAGCGAACCATGTGC-3′	[17]
IFN-γ	5′-TGGTAGCTCTGGGAAACTGAATG-3′	5′-GGCTTTGCGCTGGATCTG-3′	[18]
TGF-β	5′-CACGTGGAGCTATACCAGAA-3′	5′-TCCGGTGACATCA AAGGACA-3′	[19]

**Table 3:** Comparison of the performance data between the three periods. Values represent the mean and the standard error of the mean (SEM) for each performance parameter (average daily gain, feed intake, feed conversion ratio, and mortality rate). A total of 491,000 piglets were evaluated across 140 batches distributed in four nursery farms.

	LS Mean			SEM	*p*-Value
	CONTROL (*n* = 174,000)	TRANSITION (*n* = 105,000)	0M (*n* = 212,000)		
BW0, kg	5.58	5.47	5.44	0.050	ns
BW1, kg	17.40 ^a^	18.99 ^b^	18.72 ^b^	0.233	0.011
ADG, g/day	292.28 ^a^	300.09 ^ab^	307.45 ^b^	2.303	0.012
FCR	1.80 ^a^	1.59 ^b^	1.58 ^b^	0.029	0.005
Mortality, %	6.48%	5.10%	5.08%	0.004	0.089

^a,b^ Means values within a row with different superscripts differ. ns: not significant.

**Table 4:** Differences in bacterial species between pre-weaning and post-weaning periods. Values represent the mean relative abundance of the most predominant bacterial species identified in fecal samples. Analyses were conducted at the species level, including taxa with ≥1% relative abundance in at least one group.

Family/Genera/Species	LS Mean		SEM	*p*-Value
	PRE-WEANING	POST-WEANING		
*Desulfovibrionaceae/Bilophila*				
* B. wadsworthia*	0.135	0.022	0.016	<0.001
*Desulfovibrionaceae/Desulfovibrio*				
* D. piger*	0.007	0.008	0.002	0.021
*Lactobacillaceae/Lactobacillus*				
* L. reuteri*	0.007	0.017	0.003	0.021
*Eubacteriaceae/Eubacterium*				
* E. coprostanoligenes*	0.042	0.012	0.007	0.045
* E. biforme*	0.005	0.018	0.003	0.016
*Oscillospiraceae/Faecalibacterium*				
* F. prausnitzii*	0.170	0.100	0.013	0.017
*Oscillospiraceae/Ruminoccus*				
* R. bromii*	0.003	0.024	0.004	0.009
* R. faecis*	0.002	0.000	0.000	<0.001
*Prevotellaceae/Prevotella*				
* P. copri*	0.045	0.093	0.016	0.224
*Lachnospiraceae/Roseburia*				
* R. faecis*	0.017	0.021	0.005	0.425
*Clostridiaceae/Clostridium*				
* Cl. butyricum*	0.005	0.015	0.004	0.279
*Rikenellaceae/Alistipes*				
* A. shahii*	0.009	0.001	0.002	0.012

**Table 5:** Expression of intestinal integrity markers in piglets at day 0 (pre-weaning) and day 14 (post-weaning) during the 0M period. Values represent the mean relative expression and standard error of the mean for each target gene. Analyses included TJ proteins (occludin and zonulin) and biomarkers of intestinal inflammation (calprotectin, INF-γ and TGF-β).

Intestinal Marker	LS Mean		SEM	*p*-Value
	PRE-WEANING	POST-WEANING		
CALP_LOG2	11.129	13.090	0.804	0.227
OCLD_LOG2	8.024	7.250	0.413	0.430
ZON1_LOG2	7.373	6.857	0.463	0.673
IFNg_LOG2	15.507	10.016	0.716	0.002
TGFB_LOG2	10.347	4.817	0.409	<0.001

**Table 6:** Correlation between bacterial species showing significant differences between groups and intestinal integrity markers in piglets. The table presents Pearson’s correlation coefficients (ρ) between the relative abundance of bacterial taxa and the expression levels of TJ proteins (occludin and zonulin) and biomarkers of intestinal inflammation (calprotectin, IFN-γ and TGF-β).

Bacterial Species	Calprotectin	Occludin	Zonulin	IFNγ	TGFβ	Significance (*p*-Value)
*Eubacterium_biforme*	**−0.289**	−0.148	0.075	−0.238	0.639	0.070
*Alistipes_shahii*	−0.232	−0.207	−0.047	0.263	−0.274	ns
*Lactobacillus_reuteri*	0.161	**−0.330**	−0.052	−0.018	0.001	0.087
*Prevotella_copri*	−0.205	−0.035	0.017	0.044	0.286	ns
*Bilophila_wadsworthia*	−0.048	0.138	0.125	**0.421**	0.380	0.093
*Desulfovibrio_piger*	0.234	**0.407**	−0.179	0.343	0.085	0.032
*Eubacterium_coprostanoligenes*	0.223	−0.037	−0.120	0.145	−0.373	ns
*Roseburia_faecis*	**0.291**	0.113	−0.236	0.075	−0.064	0.069
*Faecalibacterium_prausnitzii*	−0.152	0.270	**0.446**	0.250	0.584	0.033
*Clostridium_butyricum*	0.052	0.114	0.105	0.192	-	ns
*Ruminococcus_faecis*	−0.026	−0.239	0.003	0.240	**0.828**	0.006
*Ruminococcus_bromii*	−0.249	−0.193	−0.052	−0.269	-	ns
*Bacteroides_fragilis*	**0.342**	0.277	0.029	-	−0.374	0.031

Values in bold have significant correlations. ns: not significant.

## Data Availability

All relevant data are included in the manuscript. Certain data and materials are subject to institutional or legal restrictions and may be available upon request with appropriate approval.
